# Perception of low dose radiation risks among radiation researchers in Korea

**DOI:** 10.1371/journal.pone.0171777

**Published:** 2017-02-06

**Authors:** Ki Moon Seong, TaeWoo Kwon, Songwon Seo, Dalnim Lee, Sunhoo Park, Young Woo Jin, Seung-Sook Lee

**Affiliations:** 1 Laboratory of Low Dose Risk Assessment, National Radiation Emergency Medical Center, Korea Institute of Radiological & Medical Sciences, Seoul, Korea; 2 Departments of Pathology, Korea Cancer Center Hospital, Korea Institute of Radiological & Medical Sciences, Seoul, Korea; Leibniz Institute for Prvention Research and Epidemiology BIPS, GERMANY

## Abstract

Expert’s risk evaluation of radiation exposure strongly influences the public’s risk perception. Experts can inform laypersons of significant radiation information including health knowledge based on experimental data. However, some experts’ radiation risk perception is often based on non-conclusive scientific evidence (i.e., radiation levels below 100 millisievert), which is currently under debate. Examining perception levels among experts is important for communication with the public since these individual’s opinions have often exacerbated the public’s confusion. We conducted a survey of Korean radiation researchers to investigate their perceptions of the risks associated with radiation exposure below 100 millisievert. A linear regression analysis revealed that having ≥ 11 years’ research experience was a critical factor associated with radiation risk perception, which was inversely correlated with each other. Increased opportunities to understand radiation effects at < 100 millisievert could alter the public’s risk perception of radiation exposure. In addition, radiation researchers conceived that more scientific evidence reducing the uncertainty for radiation effects < 100 millisievert is necessary for successful public communication. We concluded that sustained education addressing scientific findings is a critical attribute that will affect the risk perception of radiation exposure.

## Introduction

Public concerns about radiation exposure have intensified due to an increased amount of radiation use (e.g., for medical diagnosis and disease treatment, industrial applications, and scientific and educational uses) [1]. The Fukushima-Daiichi nuclear power plant accident in March 2011 spread great fear and anxiety about the health risks of radiation exposure, even at extremely low levels of radiation (several microsieverts), which is found in natural background levels. After the accident, severe public confusion in Korea resulted in temporary closures of schools, massive selling of masks that protect from radioactive dust inhalation, and obstinate refusal of Japanese farming products. These consequences occurred even though there were several official announcements from the Korean government that there was no evidence of substantial radioactive contamination [2]. The stigma that arises from nuclear disasters such as the atomic bombings in Japan and the Chernobyl accident has affected the overall public perception concerning radiation risks. Negative attitudes about nuclear energy adversely affect the risk perception of the beneficial uses of radiation [3, 4]. Furthermore, some people hesitate to agree to accept medical care that includes radiation use in Korea [5].

Previous studies indicated that nuclear accidents cause additional negative effects on the general public’s perception toward radiation exposure and atomic energy [6, 7]. Compared with radiation experts, lay people tend to perceive that exposure to radiation carries a greater risk of harm. This perception is not surprising given that the public generally overestimates the risk of radiation and that there is an obvious discrepancy between persons’ and experts’ perception levels [8–10].

Radiation risk estimates by the public may be seriously influenced by several factors (e.g., personal interest, related knowledge, previous experience, media coverage, social representation, communication credibility, and confidence in government) [9, 11]. Scientific evidence concerning health risks is a critical factors that affects experts’ risk perception of radiation levels < 100 millisievert (mSv), and it is often used for communication with the public.

Many studies, including INWORKS study, involving nuclear workers reported that radiation exposure at low levels (i.e., < 100 mSv) could increase the risk of cancer [12, 13]. However, other papers provided a different view: that there are uncertainties on the health effects of radiation exposure in these low doses [14–16]. For example, some studies addressing the health effects from the Three Mile Island accident showed an inconsistent risk of lung cancer and leukemia when there were low levels of radiation exposure (i.e., 0.09–0.25 mSv). These inconsistent results depended on the follow-up times and analytic methods [17, 18].

Moreover, many international authorities involved in radiation protection (e.g., the International Commission on Radiological Protection, the United Nations Scientific Committee on the Effects of Atomic Radiation (UNSCEAR), and the Nuclear Regulatory Commission) recommend that much more scientific evidence is needed to decrease the uncertainty about the radiation risk data at exposure to < 100 mSv [19–22]. Nonetheless, some radiation experts (e.g., scientists, technologists, instructors, or public communicators) have given the public non-conclusive information about the health effects of low dose radiation below 100 mSv. This incorrect information has increased societal confusion and resulted in loss of the public’s trusts [23]. Radiation experts’ risk perceptions about the health effects of radiation < 100 mSv is one of important contributing factors affecting public perception [24]. Radiation experts can reduce this confusion about the health effects of ionizing radiation if they provide scientific information that includes concrete concepts of risk expressed by the established benefits of, and damage from, radiation application.

Radiation researchers in the life sciences including biologists, epidemiologists, clinical doctors, and physicists can estimate the health effects of radiation exposure based on the experimental results and population-based observational data using scientific methods. They know that they are at a greater risk of radiation exposure due to the frequent use of artificial radiation sources for their experiments, except for epidemiologists. Since these investigators are annually take the educated in radiation safety, they have a high chance of knowing accurate information about radiation exposure risks. Therefore, it is essential to evaluate researchers’ risk perception about radiation exposure at extremely low levels (i.e., several microsieverts) to devise more reliable risk communication strategies with the public.

## Materials and methods

### Survey method

A questionnaire survey was administered during the December 2015 annual meeting of the Korean Society of Radiation Biosciences. The society’s membership includes biologists, veterinarians, epidemiologists, and medical staff who are practicing nuclear medicine, radiation oncology, and medical imaging. All members were either masters students or possessed either masters or doctorate degrees in their respective fields. They annually attend the required legal radiation education program for usage, protection, and related laws, in Korea.

**Fig 1:**
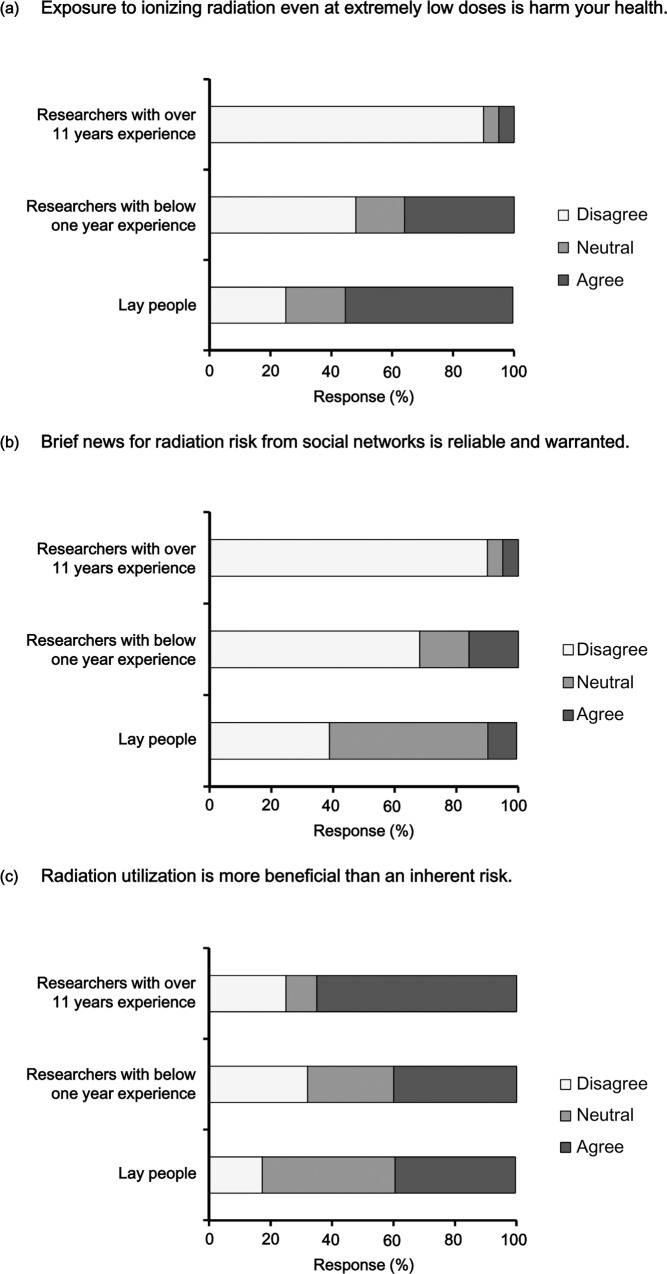
Comparison of radiation perception between research scientists and laypersons. Responses of research scientists to the following questions were recategorized and compared with previously published responses from laypersons. The three categories of responses to following were “disagree,” “neutral,” and “agree.”: (a) “Exposure to ionizing radiation even at extremely low doses (several microsieverts) might harm your health;” (b) “Do you agree that brief news about radiation risk from social networks, such as Facebook and Twitter, is reliable and warranted?;” and (c) “Radiation utilization is thought to be more beneficial than to present an inherent risk to human life.”

The questionnaire used was devised for this study by the authors and it was validated by a specialized epidemiologist and statistician for survey study from the Korean Society of Radiation Biosciences. Then, it was pilot tested by small group of radiation researchers and modified before execution as a large-scale survey. Some questions had been utilized in a previous domestic survey (see Fig 1) [2]. The questionnaire consisted of 22 main questions: It included 4 questions addressing radiation risk perception, 2 questions addressing radiation protection regulations, 6 questions addressing radiation exposure below 100 mSv, 3 questions addressing information credibility, 1 question addressing research environmental risk factors (7 sub-questions addressing perception score), and 6 questions addressing respondents’ personal information (i.e., sex, age, professional level, duration of research experience, radiation usage frequency, and warning of exposure dose) (Table 1). Responses were measured using a Likert-style scale that was scored from 1 to 7. Survey respondents were assured of the anonymity of their responses and that their results would not affect their performance in a specific study course or topic. The questionnaire is available (S1 Supporting Information). This study was approved by the institutional review board (IRB) of Korea Institute of Radiological & Medical Sciences (IRB number: K-1608-002-042). This study was conducted in accordance with the Declaration of Helsinki.

**Table 1:** Participants’ socio-demographic characteristics.

	Frequency (persons)	Percentage (%)
Total	120	100.00
Sex		
Male	64	53.33
Female	56	46.67
Age	
20–29	37	31.09
30–39	46	38.66
40–49	31	26.05
50–59	5	4.20
Professional level		
Student in master’s course	12	10.00
Researcher with a master’s degree	54	45.46
Researcher with a doctorate degree	29	24.54
Professor or principal researcher	24	20.00
Experience with radiation research		
Less than 1 year	25	21.01
1–3 years	35	29.41
4–5 years	22	18.49
6–10 years	17	14.29
11 years or more	20	16.81
Radiation use frequency		
Rarely or never	22	18.33
Two or three times/month	50	41.67
Two or three times/week	36	30.00
Everyday	12	10.00

### Definition of low dose radiation

The concept for level of radiation exposure in this study followed the recommendations of the International Commission on Radiological Protection’s (ICRP) publication 99 (2005). As a rough rule of thumb, they called referred to doses of 1 Sv, 100 mSv, 10 mSv, 1 mSv, and 0.1 mSv (100 microsieverts) as “moderately high”, “moderate”, “low”, “very low”, and “extremely low,” respectively [25]. For a clear understanding of exposure level, we described the levels with the appropriate numeric scale.

### Statistical analysis

To explore variables related to the risk perception of radiation exposure, the responses to question 11, “Exposure to ionizing radiation even at extremely low doses (several microsieverts) might harm your health” was set as the dependent variable. We set characteristics (age, sex, professional level, experience period, and radiation use frequency) as independent variables for the univariate and multiple variate linear regressions. Means and standard deviations were calculated with the answers from the scale. Spearman’s correlation analysis was used to determine the relationships between the answers to some questions and respondents’ characteristics (i.e., duration of experience in radiation research and radiation use frequency). An independent sample t-test was used to evaluate between-group differences. A one-way analysis of variance with a Tukey’s post hoc test was also used to examine differences between research scientists with ≥ 11 years of experience and scientists with < 11 years of experience. All analyses were performed using the Statistical Package for the Social Sciences version 23 (IBM, Chicago, IL), and the standard for statistical significance was set at *p* < 0.05. Mean score and standard deviation values, 95% confidence intervals (CIs), and *p* values were summarized in tables. For comparison with previously reported data regarding risk perception of lay people, our 7-point scale responses were recategorized as “disagree” for respondents who selected 1, 2, or 3, “neutral” for respondents who selected 4, and “agree” for respondents who selected 5, 6, or 7. Previously published data consisting of 5-point scale responses were transformed as “disagree” for respondents who selected 1 or 2, “neutral” for respondents who selected 3, and “agree” for respondents who selected 4 or 5. We then calculated the results as percentage values (i.e., of total numbers of respondents) for each category of responses.

## Results

### Participants’ general characteristics

One-hundred twenty of the 140 distributed surveys were returned (85.7% collection rate). Experience level and radiation use frequency were asked to assess familiarity with use of radiation in research. Approximately 50% of the respondents had less than 3 years of experience with radiation research and less than a quarter had ≥ 11 years of experience with radiation (Table 1).

### Factors associated with risk perception of extremely low level radiation exposure

The research scientists’ perceptions about radiation risk associated with extremely low levels (several microsieverts) of exposure were explored depending on the respondents’ characteristics (Table 1). Radiation research scientists perceived that the radiation risk is low (mean = 2.97, SD = 1.79). Responses were analyzed using univariate and multivariate linear regression to examine what factors were associated with the risk perception of extremely low radiation. Being a man, being aged 40–49 years, having ≥ 11 years of experience, and being a professor principal researcher were all significantly associated with the perception of radiation risk (Table 2). Radiation use frequency was not significantly associated with risk perception. Moreover, only ≥ 11 years of experience was significantly associated with the risk perception of an extremely low level of radiation after controlling for age, sex, professional level, and radiation use frequency (B = −2.456, *p* = 0.004). None of the other variables were statistically correlated with radiation risk perception.

**Table 2:** Linear regression analyses of the factors associated with responses to the sentence, “Exposure to ionizing radiation even at extremely low doses (several microsieverts) might harm your health”.

Factor	Mean (SD)	Univariate		Multivariate	
		^†^B (95% CI)	*p*-value	^†^B (95% CI)	*p*-value
Total response	2.97 (1.79)				
Sex					
Woman	3.38 (1.89)	Reference		Reference	
Man	2.58 (1.60)	-0.792 (-1.434, -0.149)	0.016*	-0.320 (-1.049, 0.410)	0.387
Age (years)					
20–29	3.43 (1.76)	Reference		Reference	
30–39	3.09 (1.78)	-0.342 (-1.115, 0.432)	0.383	-0.092 (-0.975, 0.790)	0.836
40–49	2.30 (1.73)	-1.132 (-1.984, -0.281)	0.010*	0.158 (-1.305, 1.621)	0.830
50–59	2.75 (1.50)	-0.682 (-2.507, 1.142)	0.460	1.498 (-1.015, 4.011)	0.240
Professional level					
Student in master’s course	4.00 (2.05)	Reference		Reference	
Researcher with master’s degree	3.15 (1.59)	-0.849 (-1.989, 0.291)	0.143	-0.727 (-1.879, 0.426)	0.214
Researcher with doctorate degree	2.90 (1.90)	-1.103 (-2.322, 0.115)	0.075	-0.563 (-1.930, 0.804)	0.416
Professor or principal researcher	2.18 (1.71)	-1.818 (-3.089, -0.547)	0.005*	-0.522 (-2.412, 1.368)	0.585
Experience with radiation research					
Less than 1 year	3.76 (1.92)	Reference		Reference	
1–3 years	3.00 (1.64)	-0.760 (-1.632, 0.112)	0.087	-0.596 (-1.563, 0.371)	0.224
4–5 years	3.32 (1.80)	-0.444 (-1.457, 0.569)	0.387	-0.431 (-1.552, 0.689)	0.447
6–10 years	2.94 (1.81)	-0.822 (-1.888, 0.243)	0.129	-0.670 (-1.994, 0.654)	0.318
11 years or more	1.60 (1.10)	-2.160 (-3.159, -1.161)	< 0.001*	-2.456 (-4.090, -0.822)	0.004^*^
Radiation use frequency					
Rarely or never	3.27 (1.75)	Reference		Reference	
Two or three times/month	3.23 (1.82)	-0.039 (-0.943, 0.865)	0.933	0.120 (-0.863, 1.103)	0.810
Two or three times/week	2.69 (1.80)	-0.578 (-1.525, -0.369)	0.229	-0.125 (-1.156, 0.905)	0.809
Everyday	2.10 (1.38)	-1.182 (-2.474, 0.110)	0.073	-0.004 (-1.457, 1.450)	0.996

*p < 0.05.

^†^Unstandardized coefficients in the linear regression analyses.

Adjusting factors: age, sex, professional level and radiation use frequency.

All items were rated using seven-point Likert scale: 1 (entirely disagree) to 7 (entirely agree).

CI: confidential interval; SD: standard deviation.

### Risk perception of radiation exposure during research activities

Researchers were asked some questions about specific situations (Table 3). Most researchers perceived that radiation exposure during daily life activities, including medical diagnoses, came with a very low health risk (mean = 1.92, SD = 1.09). However, they conceived that the radiation risk will significantly increase when radiation is used for research activities. The respondents perceived that radiation use in research can induce more health problems at minor or harmful levels, compared with radiation exposure in daily life (all *ps* < 0.001). A Spearman analysis showed that risk perception of radiation in research activities was inversely correlated with research experience and radiation use frequency. Specific news from the media about radioactive materials significantly changed the risk perception of research scientists. When they received news from the media, radiation risk perception increased. This response was also negatively correlated with experience. Scientists with < 1 year of experience perceived that radiation exposure in research is very dangerous after hearing brief news for radiation concerns; however, this was not true for researchers with ≥ 11 years of experience, who showed that the risk perception of radiation was not significantly altered compared with that of their daily activities (*p* = 0.019).

**Table 3:** Research scientists’ risk perception of radiation exposure using a Spearman correlation analysis.

Item	Mean (SD)	Radiobiology experience (mean (SD))						Radiation use frequency (mean (SD))				
		Less than 1 year	1–3 years	4–5 years	6–10 years	11 years or more	*Spearman's r*	Rarely or never	2–3 times/month	2–3 times/week	Everyday	*Spearman's r*
Radiation exposure in daily life is worrisome (including medical radiation exposure).	1.92	2.2	1.89	2.05	1.76	1.65	-0.204*	2.09	1.92	1.78	2.00	-0.124
	(1.09)	(1.08)	(0.93)	(1.29)	(1.20)	(1.04)		(1.06)	(0.99)	(1.22)	(1.21)	
Research activities using ionizing radiation will cause relatively minor health problems (e.g., dizziness and chest tightness), but not disease.	2.46	3.24	2.51	2.64	2.12	1.50	-0.330**	3.05	2.74	1.94	1.75	-0.297^**^
	(1.67)	(1.96)	(1.54)	(1.71)	(1.58)	(1.00)		(1.70)	(1.85)	(1.33)	(1.06)	
Radiation exposure during research activities will harm your health.	2.63	2.88	2.83	2.95	2.50	1.75	-0.201*	2.59	3.00	2.28	2.27	-0.145
	(1.79)	(1.96)	(1.44)	(1.75)	(1.71)	(1.02)		(1.53)	(1.71)	(1.61)	(1.27)	
Radiation exposure after seeing a news brief on “a small amount of radioactive material found in domestic foods” is worrisome.	3.58	4.12	3.83	3.32	3.63	2.70	-0.269**	3.27	3.98	3.44	2.82	-0.078
	(1.65)	(1.62)	(1.38)	(1.70)	(1.93)	(1.59)		(1.45)	(1.78)	(1.44)	(1.83)	

*p < 0.05.

**p < 0.01.

All items were rated using a seven-point Likert scale: 1 (entirely disagree) to 7 (entirely agree).

SD: standard deviation.

### Risk perception for the health effects of radiation exposure at <100 mSv

Researchers were asked specific questions concerning exposure at < 100 mSv (UNSCEAR’s definition of a low dose radiation range), including knowledge, interest, need for research, and communication) (Table 4). A considerable number of respondents (23.28%) selected > 5 from the 7-point score options answered, implying that exposure to extremely low levels (several microsieverts) of radiation was harmful to one’s health. This response was inversely proportional to radiation familiarity, the radiation research experience period (*r* = −0.346, *p*<0.001), and radiation use frequency (*r* = −0.218, *p* = 0.019). Respondents also conceived that they did not have much knowledge about, but have much interest in, the health effects of exposure to radiation levels <100 mSv. These responses were significantly correlated with radiation familiarity. Regardless of length of experience, all scientists strongly agreed that more biological research is necessary to reduce the uncertainty about the radiation health risks at < 100 mSv. Moreover, more than 90% of the more experienced researchers (≥ 11 years of experience; 18 of 20 respondents) and everyday radiation users (10 of 11 respondents) agreed that more biological evidence is needed. We also asked whether the radiation research scientists intend to inform lay people about the health risks of exposure to radiation levels < 100 mSv. Approximately 90% (88.8%) of the respondents who selected > 4 (the median of the 7-point scale) were willing to convey health information to lay people if they could acquire scientific evidence about radiation risk.

**Table 4:** Analysis of the awareness and necessity for scientific evidence about effects of radiation exposure at <100 mSv.

Item	Mean (SD)	Period of experience in radiobiology (mean (SD))						Radiation use frequency (mean (SD))				
		Less than 1 year	1–3 years	4–5 years	6–10 years	11 years or more	*Spearman's r*	Rarely or never	2–3 times/month	2–3 times/week	Everyday	*Spearman's r*
Exposure to ionizing radiation even at extremely low doses (several microsieverts) might harm your health.	2.97	3.76	3.00	3.32	2.94	1.60	-0.346**	3.27	3.23	2.69	2.09	-0.218*
	(1.79)	(1.92)	(1.64)	(1.8)	(1.81)	(1.1)		(1.75)	(1.82)	(1.80)	(1.38)	
Are you interested in the effects of low-dose radiation exposure < 100 mSv exposure on the human body?	3.78	3.48	3.00	3.89	3.87	5.35	0.390**	2.86	3.47	4.39	4.82	0.406**
	(1.59)	(1.53)	(1.33)	(1.29)	(1.50)	(1.39)		(1.36)	(1.36)	(1.50)	(2.04)	
How much do you know about the results of biological research on low-dose radiation exposure < 100 mSv?	4.55	3.92	4.23	4.11	5.25	5.75	0.352**	3.64	4.62	4.72	5.36	0.237*
	(1.79)	(1.85)	(1.52)	(1.63)	(1.88)	(1.62)		(2.01)	(1.51)	(1.68)	(2.25)	
The ICRP and UNSCEAR have called for research on the biological effects of low-dose radiation < 100 mSv to reduce uncertainty. Do you agree with this idea?	5.31	5.32	4.74	5.11	5.44	6.35	0.282^**^	6.36	5.23	5.13	5.27	0.150
	(1.44)	(1.22)	(1.46)	(1.23)	(1.75)	(1.04)		(1.03)	(1.57)	(1.33)	(1.45)	
Do you want to explain the biological effects of low dose radiation in a scientific manner?	4.03	4.00	3.51	4.00	4.13	4.95	0.195*	3.59	3.83	4.50	4.27	0.205*
	(1.59)	(1.32)	(1.54)	(1.33)	(1.86)	(1.73)		(1.59)	(1.46)	(1.48)	(2.15)	
If there is scientific evidence for the effects of low-dose radiation on humans, are you willing to learn about it and actively inform the people around you?	5.25	5.56	4.51	5.21	5.00	6.40	0.211*	5.18	5.02	5.31	6.00	0.123
	(1.53)	(1.23)	(1.62)	(1.27)	(1.90)	(0.68)		(1.71)	(1.52)	(1.45)	(1.41)	

*p<0.05.

**p<0.01.

All items were rated using seven-point Likert scale: 1 to 7.

SD: standard deviation; mSv: millisievert; ICRP: International Commission on Radiological Protection; UNSCEAR: United Nations Scientific Committee on the Effects of Atomic Radiation

### Radiation risk perception of research scientists compared to that of lay people

Research scientists with a short amount of experience (< 1 year) conceived that radiation exposure had a higher health risk at the level of several microsieverts (Table 2). The results were similar for the risk perception of lay people. To investigate the perception gap between the public and researchers, we compared the current results with the previous ones of lay people in Korea [2]. We recategorized the answers of some questions into three responses (“disagree,” “neutral,” and “agree.”). The percentage of respondents who agreed to be asked about “Exposure to ionizing radiation even at extremely low doses (several microsieverts) might harm your health.” was 55% for laypersons, 36% for researchers with < 1 year of experience, and 5% of for researchers with ≥ 11 years of experience. Moreover, researchers and respondents who disagreed was 25% for laypersons, 48% for researchers with < 1 year of experience, and 90% for researchers with ≥ 11 years of experience (Fig 1A). Those who disagreed with the question, “Do you agree that brief news about radiation risk from social networks, such as Facebook and Twitter, is reliable and warranted?” increased depending on experience (38.8% for laypersons, 68% for researchers with < 1 year of experience, and 90% for researchers with ≥ 11 years of experience) (Fig 1B). Many lay people and researchers with < 1year of experience agreed with the statement that “radiation utilization is thought to be more beneficial than presenting an inherent risk to human life” (39.1% of laypersons, 40% of researchers with 1 < year of experience, and 65% of researchers with ≥ 11 years of experience) (Fig 1C).

## Discussion

We hypothesized that researchers’ experience period and frequency of radiation use would affect their risk perception of radiation exposure. These radiation familiarity factors might discriminate between research scientists and lay people. A Spearman correlation analysis revealed that risk perception of radiation exposure to extremely low levels (several microsieverts) was inversely correlated with the amount of research experience. Research scientists with ≥ 11 years of experience consistently answered that radiation exposure during daily life and research activities has a low health risk. An in-depth analysis of nuclear experts revealed that the more professional knowledge and experience one has, the lower one’s radiological risk perception is [8]. In contrast, some respondents perceived that radiation exposure during research activities is riskier to one’s health than is the exposure in daily life. Interestingly, they had never received any warnings about over-exposure from a regulatory organization for health protection. This lack of warning means they had not been exposed to radiation at 25% of the average annual dose limit (5 mSv) during research activities. Given that radiation exposure can be several to tens of mSv during some medical exposures, their risk perception of radiation exposure in research activities is somewhat unexpected and thought to be not based on scientific evidence. Respondents with this irrational risk perception also recognized that they had less knowledge about the health effects of exposure to radiation < 100 mSv when compared against the more educated researchers. Therefore, our results propose that insufficient knowledge due to a lack of experience could cause an irrational risk perception and becoming more educated on the scientific evidence about radiation risk at < 100 mSv exposure could help researchers achieve a reasonable risk perception. However, we cannot exclude the possibility that more experience could make researchers less alert about radiation’s potential risks, since they had not received any warning for a long time, although they were always using it. Another explanation for the irrational response is that they perceived the risk of medical exposure more generously, since the psychological characteristics of these risks including voluntariness, and distribution of risks and benefits are different [8, 26]. Future research should examine these possibilities to analyze the risk perception of respondents for radiation exposure.

A high level of interest in the health effects of radiation exposure at < 100 mSv could indicate that research scientists do not have enough information to alleviate concerns about radiation risk. Moreover, many believed that more biological research studies should be conducted to reduce the uncertainty concerning health risks. The need for additional scientific evidence was confirmed by the intentions of the research scientists to answered questions from lay people about radiation risk. When they could acquire scientific information from experimental results, the research scientists were significantly more willing to answer the questions to the public about the radiation-induced health risks at < 100 mSv. Taken together, these results showed that more biological research studies on the health effects of radiation exposure at < 100 mSv could lead to more active communication between researchers and the public. This might also improve the credibility of these scientists’ opinions.

Although human epidemiological studies of radiation exposure have contributed to the golden standard for radiation safety, there is still uncertainty on the health effects of low dose radiation exposure (i.e., < 100 mSv). International authorities related to radiation protection such UNSCEAR and ICRP recommended that biological research on the effects of low dose radiation, accompanied with epidemiological studies with large cohorts, should be performed to reduce this uncertainty [27, 28]. Radiobiological studies combined with new methods such as systems biology can be a useful approach to estimate the health effects of low dose radiation if its impediments are overcome such as an absence of applicable radio-biomarkers and assessable model systems on human.

A comparison analysis for risk perception between laypersons and radiation researchers showed that research scientists with < 1 year of experience conceived a median risk of radiation exposure, which falls somewhere between the public and more educated researchers. This suggests that even a brief amount of radiation experience, which includes education and training, is sufficient to alter one’s risk perception. Compared with the public, research scientists have a relatively greater number of opportunities to receive scientific information for radiation’s effects. Increased opportunities to educate one’s self with scientific evidence could increase the probability that the differences between these groups will decrease. Discrepancies about the risk perception between experts and the public have previously been investigated from the viewpoint of associated risk knowledge, expressed words, confidence in the information source, social acceptance, and training and education [5, 9, 29]. Similar to our results, these studies indicated that experience-related knowledge level is one of attributes that explains the risk perception gap between scientists and the public.

Our study was limited by its relatively small sample size and did not classify respondents by their major field or specialty (e.g., biology, medicine, health physics, and epidemiology). However, this exploratory analysis of Korean radiation researchers in the life sciences clearly revealed that the risk perception of radiation exposure at less than 100 mSv is significantly associated with researchers’ experience level. Risk perception can be affected by many complicated factors including trust with the media’s news statements, personal experience, psychological acceptance, and regional customs. We should carefully consider these factors to understand risk perception. Additional scientific evidence, provided thru education, can help the public shape a more rational perception of radiation’s risks and benefits.

## Supporting information

S1 Supporting Information Questionnaire addressing the perception of low dose radiation exposure. (DOCX)
