# Supplementary material for: Perception of low dose radiation risks among radiation researchers in Korea
Source: PLoS One. 2017 Feb 6;12(2):e0171777. doi: 10.1371/journal.pone.0171777 (PMC5293274; doi:10.1371/journal.pone.0171777)
Supplement: S1 Supporting Information — (DOCX) [file pone.0171777.s001.docx]

**A Survey on the Perception of Low-Dose Radiation Exposure**

Following the Fukushima nuclear disaster in 2011, public concern about radiation exposure has been growing. Consequently, the Nuclear Safety and Security Commission are currently establishing mid- and long-term plans for biological research on low-dose radiation to secure scientific evidence for a low-dose radiation safety policy. The survey results will be used as supporting data to identify the perceptions of low-dose radiation exposure below 100 mSv among radiation researchers and thereby discover important topics for research.

December 2015, Korea Institute of Radiological & Medical Sciences

1. What is your sex?

① Male ② Female

2. What is your position?

① Student ② Master researcher (including those attending a doctorate course)
③ PhD researcher (including post-docs) ④ Professor (senior researcher)

3. What is your age?

① 20–29 Years ② 30–39 Years ③ 40–49 Years ④ 50–59 Years ⑤ 60–69 Years

4. How long have you been in radiation research fields?

① Less than one year ② Less than three years ③ Less than five years ④ Less than 10 years

⑤ 11 years or more

5. How often do you use radiation for your study?

① Every day ② 2-3 times a week ③ 2-3 times a month ④ Rarely or never

6. Have you ever received warnings or undergone special care for health protection because of the accumulated dose on the personal dosimeter (TLD or film badge) that you wear during research?

① Yes ② No

※ Please answer the following the question on a scale of **1 to 7**

7. How dangerous are the followings to the health of a researcher?

|  | ①  Very Low | ② | ③ | ④ | ⑤ | ⑥ | ⑦  Very High |
| --- | --- | --- | --- | --- | --- | --- | --- |
| Ionizing radiation exposure |  |  |  |  |  |  |  |
| Electromagnetic waves or UV ray exposure |  |  |  |  |  |  |  |
| Chemical reagent exposure |  |  |  |  |  |  |  |
| Smoking (including secondhand smoking) |  |  |  |  |  |  |  |
| A hygienic laboratory environment |  |  |  |  |  |  |  |
| LMO experimental materials |  |  |  |  |  |  |  |
| Stress from research |  |  |  |  |  |  |  |

※ Please answer the following sentences depending on the degree of agreement with a scale of

**1 (entirely disagree) to 7 (entirely agree)** (8-11)

| 8. Radiation exposure in daily life is worrisome (including medical radiation exposure). | | | | | | |
| --- | --- | --- | --- | --- | --- | --- |
| 1. ② ③ ④ ⑤ ⑥ ⑦ | | | | | | |
| 9. Research activities using ionizing radiation will cause relatively minor health problems (e.g., dizziness and chest tightness), but not disease. | | | | | | |
| ① ② ③ ④ ⑤ ⑥ ⑦ | | | | | | |
|  |  |  |  |  |  |  |
| 10. Radiation exposure during research activities will harm your health. | | | | | | |
| ① ② ③ ④ ⑤ ⑥ ⑦ | | | | | | |
|  |  |  |  |  |  |  |
| Exposure to ionizing radiation even at extremely low doses (several microsieverts) might harm your health. | | | | | | |
| ① ② ③ ④ ⑤ ⑥ ⑦ | | | | | | |
|  |  |  |  |  |  |  |
| ※ Please answer the following questions on a scale of **1 to 7** (12-16) | | | | | | |
| 12. Are you interested in the effects of low-dose radiation exposure < 100 mSv exposure on the human body? | | | | | | |
| ① absolutely no ② mostly no ③ somewhat no ④ neither yes or no ⑤ somewhat yes ⑥ mostly yes  ⑦ absolutely yes | | | | | | |
| 13. How much do you know about the results of biological research on low-dose radiation exposure < 100 mSv? | | | | | | |
| ① not at all ② very little ③ little ④ some ⑤ somewhat much ⑥ much ⑦ very much | | | | | | |
| 14. The ICRP and UNSCEAR have called for research on the biological effects of low-dose radiation < 100 mSv to reduce uncertainty. Do you agree with this idea? | | | | | | |
| ① entirely disagree ② mostly disagree ③ somewhat disagree ④ neither agree nor disagree ⑤ somewhat agree ⑥ mostly agree ⑦ entirely agree | | | | | | |
| 15. Do you want to explain the biological effects of low dose radiation in a scientific manner? | | | | | | |
| ① absolutely no ② mostly no ③ somewhat no ④ neither yes or no ⑤ somewhat yes ⑥ mostly yes  ⑦ absolutely yes | | | | | | |
| 16. If there is scientific evidence for the effects of low-dose radiation on humans, are you willing to learn about it and actively inform the people around you? | | | | | | |
| ① absolutely no ② mostly no ③ somewhat no ④ neither yes or no ⑤ somewhat yes ⑥ mostly yes  ⑦ absolutely yes | | | | | | |
| ※ Please answer the following sentences depending on the degree of agreement with a scale of **1 (entirely disagree) to 7 (entirely agree)** (17-22) | | | | | | |
| 17. Radiation risks can be controlled by current scientific knowledge. | | | | | | |
| ① ② ③ ④ ⑤ ⑥ ⑦ | | | | | | |
|  |  |  |  |  |  |  |
| 18. Radiation utilization is thought to be more beneficial than to present an inherent risk to human life. | | | | | | |
| ① ② ③ ④ ⑤ ⑥ ⑦ | | | | | | |
|  |  |  |  |  |  |  |
| 19. The average natural radiation exposure in Korea is estimated to be 3.08 mSv/yr (KINS, 2009). The safety regulations on radiation exposure for radiation workers (below 50 mSv/yr, and below 100 mSv/5 yr) are appropriate. | | | | | | |
| ① ② ③ ④ ⑤ ⑥ ⑦ | | | | | | |
|  |  |  |  |  |  |  |
| 20. Information on radiation exposure provided by the government is reliable. | | | | | | |
| ① ② ③ ④ ⑤ ⑥ ⑦ | | | | | | |
|  |  |  |  |  |  |  |
| 21. Radiation exposure after seeing a news brief on “a small amount of radioactive material found in domestic foods” is worrisome. | | | | | | |
| ① ② ③ ④ ⑤ ⑥ ⑦ | | | | | | |
|  |  |  |  |  |  |  |
| 22. Do you agree that brief news about radiation risk from social networks, such as Facebook and Twitter, is reliable and warranted? | | | | | | |
| ① ② ③ ④ ⑤ ⑥ ⑦ | | | | | | |

**Thank you for your time and attention.**
